# Introduction to the mini‐symposium “molecular neuropathology of meningioma”

**DOI:** 10.1111/bpa.13055

**Published:** 2022-02-25

**Authors:** Felix Sahm, Christian Mawrin

**Affiliations:** ^1^ Department of Neuropathology University Hospital Heidelberg Heidelberg Germany; ^2^ CCU Neuropathology DKTK Heidelberg Germany; ^3^ DKFZ Heidelberg Germany; ^4^ Department of Neuropathology University Hospital Magdeburg Magdeburg Germany

**Keywords:** meningioma

## Abstract

Progress of molecular meningioma characterization (*courtesy of Ralf Ketter, Homburg, Germany).

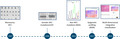

While meningiomas represent the most frequent primary intracranial tumor and, therefore, play an important role in daily clinical practice for neurosurgeons, neurooncologists, and neuropathologists, this tumor type has not been covered within the well‐recognized format of a mini‐symposium in *Brain Pathology*. Now, this gap is going to be filled, and the timing cannot be better. Both the understanding of the molecular alterations acting in meningiomagenesis, as well as the classification of the tumors by combining conventional histological features with a site of tumor growth, somatic mutations, and epigenetic characteristics has expanded our understanding of the biological landscape of this tumor previously regarded as “boring” to investigate and simple to treat. This evolution is reflected by the just recently updated WHO classification of brain tumors which includes molecular features as an important layer to classify and grade meningiomas [1] (Table 1).

**TABLE 1:** Current WHO classification of meningioma and frequently associated molecular alterations

Meningioma subtype	CNS WHO Grade	Frequent molecular alteration	Typical Methylation group (14)
(Meningioma, NOS)			
Meningothelial	1	*AKT1/TRAF7, SMO*	Ben‐2
Fibrous	1	*NF2*, 22q del	Ben‐1
Transitional	1	*NF2*, 22q del	Ben‐1
Psammomatous	1	*NF2*, 22q del	Ben‐1
Angiomatous	1	Trisomy 5	Ben‐3
Microcystic	1	Trisomy 5	Ben‐3
Secretory	1	*KLF4/TRAF7*	Ben‐2
Lymphoplasmacyte‐rich	1		(None)
Metaplastic	1	Trisomy 5	Ben‐3
Chordoid	2		Various
Clear cell	2	*SMARCE1*	Separate group
Rhabdoid	2	*BAP1*	mal (if “true” rhabdoid)
Papillary	2	*PBRM1*	mal (if “true” papillary)
Atypical	2		Enriched for int‐A/int‐B
Brain invasive	2		
Anaplastic	3	*CDKN2A/B, TERT*	mal

Initiated with the description of the loss of chromosomal material on 22q [2] and the identification of *NF2* alterations in about 50% of sporadic meningiomas in the 1990s [3], the field got boosted by the identification of several recurrent somatic mutations taking advantage of next‐generation sequencing (NGS) techniques, resulting in the denomination of a non‐*NF2* driven meningioma group [4]. Besides mutations, genome‐wide methylation profiling studies proposed epigenetic subtyping with clinical relevance [5]. For the first time, classification systems integrating various layers of information, for example, morphological, molecular, and clinical data were established, providing a reproducible system for risk assessment to the patients and clinicians. The most recent include refinements and extensions incorporating large data sets derived from genetics, epigenetics, and proteomics [6, 7], while the practical use of these classification systems is subject to further evaluation (Figure 1).

**FIGURE 1:**
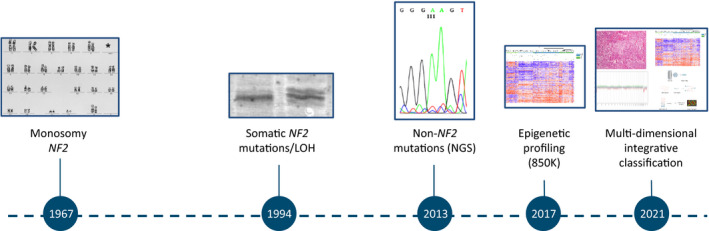
Progress of molecular meningioma characterization (*courtesy of Ralf Ketter, Homburg, Germany)

The current mini‐symposium aims to harness the molecular findings in meningioma for more practical aspects. The series is opened with a review article written by Norbert Galldiks and colleagues, summarizing the state‐of‐the‐art in the rapidly evolving field of radiogenomics. Preoperative assessment based on sophisticated image analyses using artificial intelligence approaches which address molecular characteristics might help to adjust operative strategies and reduce perioperative morbidity. Additionally, radiogenomic assessment of recurrent meningiomas may guide the decision for the optimal treatment strategy, that is, the decision between watch and wait strategy, irradiation including gammaknife or radiosurgery, and re‐operation.

This review is followed by a comprehensive study of Berghoff et al. which evaluates the practical use of methylation profiling compared to mutational analysis in a large series of clinically well‐characterized meningiomas. This data shows that both DNA methylation classification and panel‐based‐targeted mutational analysis improve the prognostic assessment and the identification of potential molecular alterations for targeted personalized therapy in meningiomas.

The paper of Berghoff and colleagues raises the question of how targeted molecular testing can be incorporated into neuropathological diagnostic. Therefore, the following article (Mawrin et al.) introduces a concise, time, and cost‐efficient NGS panel which is sufficient to detect relevant somatic mutations. The proposed panel covers all genetic alterations essential for exact classification (for instance, *KLF4*/*TRAF7* for secretory meningiomas, *SMARCE1* for clear cell meningiomas, or *BAP1* in many rhabdoid meningiomas. Moreover, prognostically relevant alterations (*TERT* promoter mutations or loss of *CDKN2A*/*B*) can be detected as well. This amplicon‐based targeted meningioma panel might accelerate the introduction of genomic characterization in daily routine work.

Finally, a review with a special focus on brain‐invasive meningiomas covers the recent knowledge about the molecular mechanisms driving this process. While brain invasion has been established as a criterion for grade 2 meningioma, the relevance for future treatment decisions and recurrence rate estimation has been subject to discussion [8].

We hope that this mini‐symposium will stipulate further research into the biological underpinnings of meningiomas and their diagnostic or even therapeutic application.

## CONFLICT OF INTEREST

There are no conflicts of interest to declare.

## AUTHOR CONTRIBUTIONS

Felix Sahm: writing, artwork. Christian Mawrin: concept, writing, artwork.

## Data Availability

Does not apply.
